# Predictors and Changes of Self-Perceived Burden Among Stroke Survivors: A 3-Month Follow-Up Study

**DOI:** 10.3389/fneur.2020.00742

**Published:** 2020-07-24

**Authors:** Yuanyuan Wei, Xiaoran Ren, Xiangni Su, Xianni Wang, Yan Hua, Yu Chen, Ruijie Shi, Pei Shao, Hongjuan Lang, Chunping Ni

**Affiliations:** ^1^School of Nursing, The Fourth Military Medical University, Xi'an, China; ^2^Leshan Retired Cadre Sanatorium, Sichuan Military Region, Leshan, China; ^3^Department of Nursing, Shaanxi Traditional Chinese Medicine Hospital, Xi'an, China; ^4^Tangdu Hospital, The Fourth Military Medical University, Xi'an, China

**Keywords:** longitudinal study, self-perceived burden, rehabilitation, stroke, stroke patients

## Abstract

**Background and Purpose:** Patients' self-perceived burden (SPB) is associated with distress, which has a potentially negative influence on disease rehabilitation and quality of life. Stroke represents a significant health and social burden. The aim of the study was to assess, compare, and identify predictors of SPB in stroke survivors during the first 3 months post-stroke.

**Methods:** A prospective longitudinal study was used. Consecutive stroke inpatients were recruited from the neurology department of three general hospitals in Xi'an, China. Patients were surveyed using the Self-perceived Burden Scale (SPBS) on the fourth day of admission (Acute phase, Time 1, T1) and 1 month (Time 2, T2) and 3 months (Time 3, T3) post-stroke.

**Results:** Considerable burden was experienced by 84.15–91.50% of patients in the first 3 months post-stroke. The mean score of physical burden was the highest. Over time, physical, emotional, and economic burden all declined. The following characteristics had significant association with increased patient SPB at T1, T2, and T3: age, self-evaluated economic pressure, comorbidity, and functional status (*P* < 0.01). Patients' knowledge about stroke was only significantly associated with SPB at T3 (*P* < 0.01).

**Conclusions:** Patients experienced a high degree of SPB in the early stage after stroke. Addressing the characteristics and predicting factors as well as the development of a targeted intervention for SPB may improve survival and post-stroke disability.

## Introduction

Stroke is a major cause of long-term disability worldwide (1). It is the second commonest cause of death and the leading cause of adult disability in China (2). The Chinese National Stroke Registry reported that stroke occurred in more than 7 million Chinese people in 2011, and many survivors experienced persistent difficulty with daily tasks (3). The suffering caused by post-stroke residual disability has a devastating effect on the daily lives of patients and their families. Its associated health care expenditures are enormous to both the family and the national health care system (1, 4, 5). Stroke survivors, especially those with a disabling condition, regularly rely on the family's support to meet the demands of their daily lives. For some patients, the receiving care can lead to the sense of having become “a burden to others.” This sense of burden was referred to as “self-perceived burden” (SPB) (6).

SPB is defined as “empathic concern engendered from the impact on others of one's illness and care needs, resulting in guilt, distress, feelings of responsibility, and diminished sense of self” (6). McPherson found that medium to high levels of SPB were mentioned by 70.2% of stroke patients (7). In Zou's study, 91.54% of stroke patients with hemiplegia had SPB (8). The SPB is associated with patients' sociodemographic and clinical characteristics. It might differ among patients with different family financial conditions, medical expenses, functional status, and social supports (9, 10). Patients' SPB has a negative impact on their quality of life (QOL) (7). It is worth noting that patients with higher levels of SPB were less likely to seek help from others (6). The SPB may threaten individuals' coping and continued progress following stroke. Most studies, however, focused on the burden of caregivers of stroke survivors (11) or were cross-sectional studies (8–10). Despite existing evidence that SPB experienced by stroke patients affects stroke rehabilitation, emotional and mental health, and QOL, studies on the SPB of patients in the acute and recovery phase shortly after stroke are inadequate. Thus, this longitudinal study aimed at determining the levels of SPB experienced by stroke survivors during the first 3 months post-stroke and identifying influencing factors of SPB.

## Methods

### Design and Participants

Consecutive acute stroke inpatients were recruited from the neurology department of three general hospitals in Xi'an of China from January to September 2017. In this study, acute stoke included hemorrhagic and ischemic stroke. Inclusion criteria were being diagnosed with the acute stroke confirmed by CT or MRI examination, age 18 years or older, and being willing to participate in the study and follow-up after discharge. Exclusion criteria included serious complications such as severe heart, liver, and kidney diseases; depressed level of consciousness post-stroke, brain injury, brain tumor, and/or dementia; other traumatic events during the past 6 months; having any obvious cognitive disabilities or psychiatric illness; suffering from deafness, aphasia, or other language barriers; or being unable to follow up after discharge.

### Measures

#### Sociodemographic and Clinical Variables

Participants completed a demographic profile that included information on patient's age, gender, marital status, level of education, category of medical expenses, self-evaluated economic pressure, family monthly income, time and course of stroke, medication history and type of stroke, and self-evaluated knowledge about stroke.

#### Self-Perceived Burden Scale (SPBS)

The SPBS (6) is a 10-item self-report measure. It includes three subscales: physical burden (five items), emotional burden (four items), and economic burden (one item). It uses a five-point Likert scale to indicate the degree of SPB experienced. Higher scores indicate greater perceived burden. According to the total sum score, patients' SPB was divided into three levels. The score of mild, moderate, and severe SPB is 20–30, 30–40, and ≥40, respectively. Wu and Jiang translated and tested it in patients with cancer, and the results of their study showed that the Chinese version of SPBS had acceptable internal consistency with a Cronbach's alpha of 0.91 for the SPBS (12).

#### Barthel Index

Barthel Index (BI) was used to measure patient's ability to perform activities of daily living (ADL). It has 10 items, each scored from 0 to 15. Total scores range from 0 (total dependence) to 100 (total independence) (13). A higher BI indicates a higher level of ADL. For a better interpretation, results have been grouped into four categories based on BI scores: severe disability (BI ≤ 40), moderate disability (BI = 41–60), mild disability (BI = 61–99), and no disability (BI = 100). BI is widely used for stroke patients in China (13). The scale has a good reliability with a Cronbach's alpha of 0.96.

### Procedure

Interviews were conducted on the fourth day of patient's admission (acute phase of stroke, Time 1, T1) and 1 month (Time 2, T2) and 3 months (Time 3, T3) following discharge. The participants were contacted by telephone, by WeChat platform, or personally, and invited to take part in the follow-up investigation. Baseline characteristics were collected at Time 1, and the SPBS was assessed at three time points. The researcher read the questionnaires and wrote down the answers of participants.

### Statistical Analysis

Epidata 3.0 was used to input data. SPSS 19.0 software was used to generate descriptive and inferential statistics. Sociodemographic and disease-related data and the scores of SPBS were described by means, standard deviations, and frequencies using Student's *t*-test or one-way ANOVA. The independent contributors of SPB were examined by stepwise multiple linear regression analyses. The total sum score of SPBS was the dependent variable, while sociodemographic and disease-related data were entered as the independent variables. Two-tailed significance tests were performed with a *P*-value of 0.05 as the significance level.

## Results

### Sociodemographic and Clinical Characteristics of Participants

A total of 363 stroke patients who met eligibility criteria agreed to participate in the study. At 3 months post-stroke, 328 patients completed the questionnaires. Six patients died, and 29 patients did not respond or declined to participate. A non-response analysis revealed no significant differences between participants and drop-outs (*P* > 0.05).

Of the 328 participants, there were 232 males and 96 females. Participants ranged in age from 26 to 89 years [mean (SD), 62.58 (11.37)]. A total of 318 patients (97.0%) suffered from various degrees of disability of ADL. There were 18.0, 61.3, and 17.7% patients who were mildly, moderately, and severely disabled, respectively.

### Patients' SPB and Its Change Over Time

Considerable burden was reported by 91.50% of stroke patients at T1 and by 90.55 and 84.15% of stroke patients at T2 and T3. At T1, T2, and T3, 56.03, 38.11, and 29.26% of patients were under moderate to severe SPB, respectively. Of the three dimensions of SPBS, emotional burden scored the highest at T1, and physical burden scored the highest at both T2 and T3 assessments, with mean total scores of >2.5 points (Table 1). The average level for the SPBS total and for its three dimensions declined significantly between T1, T2, and T3.

**Table 1:** Patients SPB at different time points 3 months after stroke (*n* = 328).

**Variable**	**Acute phase of stroke**	**One month post-stroke**	**Three months post-stroke**	***F***	***P***
	**Mean (SD)**	**Mean (SD)**	**Mean (SD)**		
Physical burden	3.08 (0.76)	2.74 (0.68)	2.67 (0.69)	244.08	0.00
Emotional burden	3.09 (0.79)	2.68 (0.72)	2.33 (0.80)	496.05	0.00
Economic burden	3.03 (1.56)	2.51 (1.14)	2.47 (1.14)	104.58	0.00
Total burden	3.08 (0.70)	2.69 (0.62)	2.52 (0.66)	567.28	0.00

**Figure 1:**
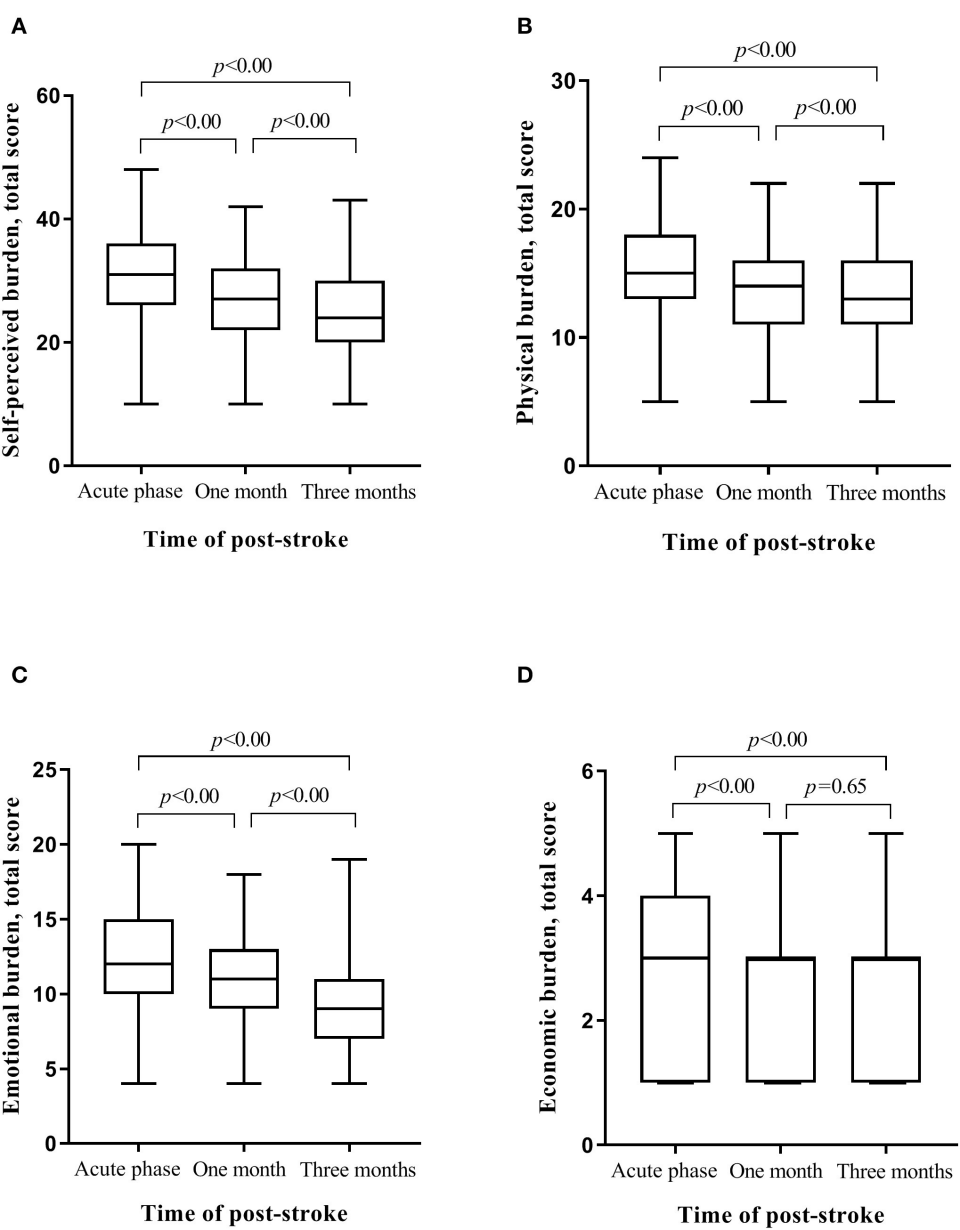
Changes of SPBS total and its three dimensions in the first three months after stroke; *P* stands for the comparison of scores of SPBS total and its three dimensions at different time. **(A)** Changes of SPBS total. **(B)** Changes of physical burdern. **(C)** Changes of emotional burden. **(D)** Changes of economic burden.

Compared with the score at T1, the total score of SPBS at T2 and T3 was reduced by 0.38 (95% confidence interval: 0.34–0.42) and 0.56 (95% confidence interval: 0.51–0.61), respectively (*P* < 0.05). The total score of SPBS at T3 decreased 0.18 (95% confidence interval: 0.15–0.20) from that of T2 (*P* < 0.05) (Table 1). Compared to the average level of SPBS' three dimensions between any two survey points, only the scores of economic burden at T2 and T3 had no statistically significant difference (*P* > 0.05). Figure 1 displays the changes of average level for the total SPBS and for its three dimensions.

### Predictors of Stroke Patients' SPB

Table 2 illustrates scores of SPB at three time points of post-stroke by different sociodemographic and clinical characteristic groups. There was a statistically significant association of age, gender, educational level, self-evaluated economic pressure, functional status, and co-occurring chronic diseases on SBP scores (*P* < 0.05). In addition, the scores of SPB at 1 month and 3 months post-stroke were different with patients' self-evaluated knowledge about stroke. To avoid the interaction of the above factors, stepwise multiple regression analysis was conducted to determine independent predictors of SPB. The following characteristics were found to have significant association with patients' greater SPB at T1, T2, and T3: age, self-evaluated economic pressure, co-occurring chronic diseases, and functional status. Patients' self-evaluated knowledge about stroke was only significantly associated with SPB at T3 (Table 3).

**Table 2:** Comparison of post-stroke SPB by sociodemographic and clinical characteristics (*n* = 328, $\bar{x}$ ± s).

**Characteristics**	***n***	**Acute phase of stroke**			**One month post-stroke**			**Three months post-stroke**		
		**Score of SPB**	***F*/*t***	***P***	**Score of SPB**	***F*/*t***	***P***	**Score of SPB**	***F*/*t***	***P***
Age (years)			9.17	0.00		−8.35	0.00		−8.04^a^	0.00
<60	125	26.70 ± 6.92			23.63 ± 5.79			21.75 ± 5.74		
≧60	203	33.26 ± 5.87			28.96 ± 5.50			27.25 ± 6.17		
Gender			−3.45	0.00		−2.70	0.01		−2.85	0.01
Male	232	29.91 ± 7.00			26.34 ± 6.03			24.50 ± 6.36		
Female	96	32.81 ± 6.75			28.34 ± 6.30			26.74 ± 6.84		
Marital status			0.63	0.53		0.59	0.56		0.29	0.78
Having a spouse	321	30.73 ± 7.81			26.90 ± 6.19			25.14 ± 6.59		
No spouse	7	32.43 ± 7.81			28.29 ± 5.44			25.86 ± 6.20		
Educational level			6.44	0.00		7.31	0.00		7.21	0.00
Elementary school	88	32.47 ± 6.73			28.38 ± 6.25			26.82 ± 7.01		
High school	199	30.62 ± 6.96			26.89 ± 5.98			25.02 ± 6.13		
College or above	41	27.80 ± 7.16			24.00 ± 5.98			22.22 ± 6.70		
Medical expenses			−1.18	0.24		−0.93	0.35		−1.24	0.22
Medical insurance	314	30.67 ± 7.04			26.86 ± 6.14			25.06 ± 6.52		
At one's own expense	14	32.93 ± 7.01			28.43 ± 6.93			27.29 ± 7.46		
Self-evaluated economic pressure			19.13	0.00		19.96	0.00		17.74	0.00
Low	86	28.17 ± 6.84			24.67 ± 5.90			22.87 ± 6.18		
Moderate	109	31.40 ± 6.48			27.40 ± 5.53			25.66 ± 5.88		
High	133	33.43 ± 6.64			29.43 ± 6.01			27.65 ± 6.64		
Functional status			15.19	0.00		30.58	0.00		39.95	0.00
Severe disability	58	34.24 ± 5.78			34.22 ± 5.59			31.00 ± 6.60		
Moderate disability	201	31.22 ± 6.75			27.65 ± 5.75			26.25 ± 5.98		
Mild disability	59	26.39 ± 7.03			22.54 ± 5.04			21.24 ± 4.56		
Independence	10	27.20 ± 6.16			22.55 ± 5.91			20.80 ± 5.09		
Times of stroke			−0.48	0.63		−1.05	0.29		−1.42	0.16
1	192	30.60 ± 7.07			26.63 ± 6.00			24.72 ± 6.14		
≥2	136	30.98 ± 7.02			27.35 ± 6.40			25.76 ± 7.11		
Co-occurring chronic diseases			19.36	0.00		18.98	0.00		21.45	0.00
0	32	28.41 ± 6.08			24.50 ± 5.33			22.50 ± 5.21		
1	118	28.77 ± 6.17			24.95 ± 5.02			22.73 ± 4.90		
2	88	29.80 ± 6.86			26.72 ± 5.96			25.31 ± 6.44		
≥3	90	35.16 ± 6.76			30.59 ± 6.42			29.12 ± 7.13		
Stroke knowledge			1.99	0.14		6.77	0.00		9.81	0.00
Rich	6	25.33 ± 13.57			22.33 ± 11.62			20.00 ± 9.32		
Normal	93	31.22 ± 6.94			26.79 ± 5.88			25.03 ± 6.24		
Deficit	229	30.72 ± 6.84			32.23 ± 7.17			32.45 ± 8.50		

**Table 3:** Factors related to post-stroke SPB (*n* = 328).

**Variable**	**Acute phase of stroke**				**One month post-stroke**				**Three months post-stroke**			
	***B***	**β**	***t***	***P***	***B***	**β**	***t***	***P***	***B***	**β**	***t***	***P***
Age	−4.48	−0.31	−6.04	0.00	−4.72	−0.33	−6.53	0.00	−2.80	−0.21	−4.68	0.00
Self-evaluated economic pressure	2.33	0.28	6.01	0.00	2.65	0.32	6.60	0.00	1.70	0.22	5.25	0.00
Co-occurring chronic diseases	1.26	0.17	3.98	0.00	1.55	0.22	4.99	0.00	1.72	0.25	6.06	0.00
Functional status	−1.66	−0.16	−3.62	0.00	−1.93	−0.19	−4.04	0.00	−2.98	−0.35	−8.09	0.00
Stroke knowledge									3.51	0.13	3.08	0.00

## Discussion

### Stroke Patients' SPB and Its Change Over Time

The current study found that a significant proportion of patients experienced SPB at 3 months post-stroke. The percentage found in the acute phase of stroke in our study is higher than that in the other studies on breast cancer patients and chronic obstructive pulmonary disease patients (14, 15). A previous study showed that stroke survivors' perceptions of self-rated health are multifactorial, comprising physical, psychological, and social components (16). The possible reason for the current finding is that many survivors in the acute phase remain physically or cognitively impaired, and the high incidence of disability and mortality might increase their SPB, especially physical burden.

In contrast with patients' SPB in the acute phase of stroke, the rate of overall burden at 1 month post-stroke did not decline, but the percentage of moderate to severe burden decreased by 17.92%. At 3 months post-stroke, the level of overall SPB decreased and the proportion of patients with moderate to severe SPB also declined. The possible reason is that, after 1 to 3 months of treatment and rehabilitation, the physical problem of some patients was partially relieved, but has not been resolved. Of the three dimensions of SPBS, the most important proved to be physical burden. The finding may be related to patients' attention to the physical function rehabilitation rather than medical costs shortly after stroke. In the current study, the relatively small decline (0.04%) was detected in the prevalence of economic burden between 1 and 3 months post-stroke. Compared to the acute phase and 1 month post-stroke, patients' economic burden rose from third to second place at 3 months post-stroke. The possible reason is that most discharged patients were still required to take drugs and rehabilitation, and the long-term treatment resulted in a heavy economic burden. These findings indicate the multidimensionality of SPB and highlight the potential usefulness of planned interventions among stroke survivors in the early stage after stroke.

### Associations and Predictors of Considerable Burden in the Acute Phase, 1 Month, and 3 Months After Stroke

Several survivor characteristics were associated with the SPB at T1, T2, and T3. One of the important findings of this study is the predictive value of age on SPB. The level of SPB increased proportionally to age. Patients over 60 years old were found to have high SPB, a finding consistent with a previous study (17). The result may due to elderly patients' serious medical conditions and poor self-care ability.

In the current study, economic pressure was found to have a significant association with SPB post-stroke. A heavier self-evaluated economic pressure was correlated to higher SPB among patients. This finding was in accordance with previous results (10). Stroke has a significant impact on health care expenditure. In 2004, the average fee for stroke admission was 6356 RMB (USD 1000.94), which was two times the annual income of rural residents (18). In this study, most patients with high economic pressure were peasants and ordinary workers. The low-income level of this population may have led them to lose confidence in treatment and rehabilitation. The interruption of treatment would negatively affect patients' psychology and physiology, thus forming a vicious cycle. Accordingly, SPB might be increased.

This study also investigated the extent to which different co-occurring chronic conditions contribute to the increase of SPB. Stroke survivors with comorbid chronic diseases such as diabetes and coronary heart disease were found to experience severe SPB. It has been suggested that co-occurring chronic diseases among stroke survivors increase the level of disability (19). A previous study has reported that the cost of treating post-stroke is highest during the first 3 months after stroke onset, which is also considered as a critical period for the occurrence of disability (20). Disability and health care expenditures from stroke and co-occurring diseases could further increase patients' SPB. The result indicates that medical staff should not only popularize the stroke knowledge to patients but also guide them to actively prevent and treat other chronic diseases. At 3 months post-stroke, the negative influence on SPB of disease knowledge lack also proved this finding.

The ability of self-care is one of the significant predictors affecting patients' SPB (15, 21). The current study found that stroke patients with lower ADL experienced more severe SPB in the first 3 months after stroke. Post-stroke disability is a great challenge to patients, their families, and the society. It contributed 4.5% of disease-adjusted life years (DALYs) from all causes (1, 22). In the current study, 97.0% patients had various degrees of dependency in the acute phase of stroke. Disability caused by stroke could impair patients' ability of self-care and influence their daily work. This not only increased patients' worry and guilt but also affected their psychological adjustment, compliance with treatment, and perspective in life. Accordingly, SPB would also increase. The finding indicates that early intervention would be helpful to improve patients' ability of self-care and reduce their SPB.

The current study also found that patients' SPB had a tendency to reduce with the level of educational background. A possible reason is that patients with high-level education had more resources and a better capacity to receive the information about stroke treatment and rehabilitation compared to those with low-level education (23). Female stroke patients were more likely to experience higher SPB than male patients. The difference of educational background and gender in stroke patients' SPB needs further study.

This study contributes to our understanding of longitudinal change and predictors of patients' SPB during the first 3 months after stroke, which provides a framework for early interventions that may alleviate certain factors influencing patients' burden such as insufficient knowledge and disability in ADL performances. Timely recognition of and attention to patients' burden and its associated factors as well as the development of a targeted intervention in the early stage of stroke might help them decrease SPB and improve QOL.

There are several limitations to the study. First, the participants were recruited from three general hospitals in one city, which might limit representation of all stroke patients in other cities in China and internationally. Second, the study evaluated patients' SPB during the first 3 months after stroke, which cannot provide evidence of long-term burden of stroke patients. Third, the study did not screen the depression of stroke survivors. Depression is the most common psychological disorder reported in stroke survivors (24), which might have a significant correlation with SPB. Hence, multicenter longitudinal studies are needed to further investigate possible predictors of patients' burden post-stroke.

## Conclusion

This study extends knowledge by providing the evolution of patient SPB in the early stage after stroke. Most patients experienced a high degree of SPB during the first 3 months post-stroke. Physical burden was the dominant burden in this phase. Over time, physical, emotional, and economic burden all declined. Patients' burden was predicted by having heavier economic pressure, comorbidity, being in poor functional status, and increase in age. Addressing the characteristics and predicting factors as well as the development of a targeted intervention for SPB may improve survival and post-stroke disability.

## Data Availability Statement

The datasets analyzed in this article are not publicly available. Requests to access the datasets should be directed to pingchunni@163.com.

## Ethics Statement

The studies involving human participants were approved by the 3 hospitals selected for study. The patients/participants provided their written informed consent to participate in this study.

## Author Contributions

CN and HL conceived and oversaw study. XW, YH, YC, RS, and PS performed data collection. YW and XS performed statistical analysis. YW and XR wrote manuscript. All authors contributed to the article and approved the submitted version.

## Conflict of Interest

The authors declare that the research was conducted in the absence of any commercial or financial relationships that could be construed as a potential conflict of interest.
